# The risk patients with AGHD have of developing CVD^☆^

**DOI:** 10.1016/j.ijcrp.2023.200221

**Published:** 2023-10-13

**Authors:** Eisha Javed, Maha Zehra, Naz Elahi

**Affiliations:** Department of Medicine, Dow Medical College, Karachi, Pakistan

**Keywords:** Pituitary disorder, Growth hormone, Cardiovascular disease, Endocrinology

## Funding information

None to declare.

## Competing interest statement

None to declare for any author.

Adult Growth Hormone Deficiency (AGHD) is an endocrine disorder that is characterized by a decreased level of secretion of Growth Hormone, from the anterior pituitary gland in the diencephalon of the brain. Approximately 6000 new cases of AGHD are diagnosed annually in the United States, less than 20 % of which represent early diagnosis in childhood [1]. This disease can be caused by congenital pituitary developmental defects, problems in hypothalamic GHRH production or release, and genetic mutation. It can also be caused by injuries to the Central Nervous System, which can be caused by tumors, trauma, radiation, and inflammatory diseases [2]. The patients suffering from AGHD have a poor quality of life, reduced life expectancy, and an increased risk of developing Cardiovascular (CV) disorders [3].

It has been proven that growth hormone plays a significant role in regulating the development of the heart, and its vascular endothelium, which helps the heart maintain its normal structure and function [4]. Deficiency of this hormone may result in CV developmental abnormalities, including decreased ventricular mass, reduced cardiac output, unfavorable alternations in lipid profile, and diminished diastolic and systolic function [5].

According to a recent study conducted by Kim et al., the CV mortality rate in adults diagnosed with AGHD was approximately twice that of those not suffering from the disease, proving its effects on CV health [6]. This can be due to the fact that the disease is characterized by increased oxidative stress, elevated fibrinogen, elevated Plasminogen Activator Inhibitor-1 activity, and higher than normal levels of low-density proteins (LDL) than high-density proteins (HDL) [7]. All of these factors lead to a higher risk of developing atherosclerosis and hypertension, both of which increase the chances of developing CV disease. In addition to this, a study conducted by Maison et al. which analyzed the life expectancy of patients with hypopituitarism, has also suggested that patients afflicted with AGHD have a fifty percent higher chance of vascular mortality [8].

In recent years, Recombinant human growth hormone replacement therapy (RGHRT) has been used to treat patients of AGHD, in order to improve their quality of life. In this treatment, synthetic growth hormone injections are administered subcutaneously and are generally considered safe, and effective. A research investigation conducted to evaluate the efficacy of RGHRT has suggested that the treatment with GH was associated with a significant improvement in cardiac structure and function [8]. Possible reasons for this can be a decrease in the atherogenic ratio, improving systolic function, and standardizing cardiac mass [9]. Furthermore, a long-term 15-year prospective research verified the beneficial effect of this treatment on lipid profile, which demonstrates one of the many benefits of RGHRT therapy in AGHD patients [10]. However, these injections have undeniable side effects, including edema, arthralgia, and paresthesia, which raise questions regarding safety [10]. Therefore, researchers need to take steps to develop newer treatments, with less dangerous side effects, in order to help AGHD patients.

Furthermore, many cases of AGHD are not diagnosed in childhood, which highlights the importance of raising awareness about this condition, as it not only impacts the patients’ memory and mood but also has detrimental effects on their CV function. Doctors should encourage patients to make changes to their lifestyle to boost Human Growth hormone naturally, such as exercising and taking arginine supplements [11]. These methods could help decrease the CV risk in these patients, reducing the risk of mortality.
